# Challenges in managing, sustaining, and assessing closed point of dispensing sites: Findings from a qualitative study

**DOI:** 10.1371/journal.pone.0271037

**Published:** 2022-07-28

**Authors:** Terri Rebmann, Kyle Foerst, Rachel L. Charney, Rachel L. Mazzara, Jessica Sandcork

**Affiliations:** 1 Institute for Biosecurity, College for Public Health and Social Justice, Saint Louis University, St. Louis, MO, United States of America; 2 Saint Louis County Department of Public Health, St. Louis, MO, United States of America; 3 Division of Pediatrics, School of Medicine, Saint Louis University, St. Louis, MO, United States of America; Drake University College of Pharmacy and Heath Sciences, UNITED STATES

## Abstract

Most U.S. public health agencies rely upon closed points of dispensing (PODs) to aid in medical countermeasure (MCM) distribution. However, few studies have focused on how to assess closed POD preparedness and none have examined best practices for managing sites once they have been recruited. This study involved qualitative interviews with U.S. disaster planners to elucidate their approaches and challenges to managing, sustaining, and assessing existing closed POD sites. In all, 16 disaster planners participated. Common management practices included frequent communication with sites, providing formal and informal training, and assisting with POD exercises. Very few jurisdictions reported doing formal assessments of closed POD sites. The largest challenges identified were staff turnover and keeping sites engaged, sometimes leading to sites voluntarily withdrawing or needing to be removed from being a closed POD. Frequent communication and building partnerships with closed POD site personnel were recommended to maintain and sustain existing sites. Formal and informal assessments will provide assurance of deployment readiness. Closed POD management is a challenging, but essential process to ensure readiness to deploy. Practices outlined by this study can be implemented to enhance closed POD network management at other jurisdictions. This should increase the ability to distribute MCMs rapidly during a future event, contributing to stronger community resilience. Public health officials should continue expanding and improving closed POD networks to enable MCM delivery and minimize morbidity and mortality related to mass casualty events.

## Introduction

Biological disasters, both natural and man-made, can pose significant threats to the population, as well as some unique challenges for public health officials. In order to effectively mitigate morbidity and mortality during these events, medical countermeasures (MCMs) will need to be dispensed rapidly throughout the community. This has been particularly relevant during the current COVID-19 pandemic and vaccine distribution planning. While various mass dispensing modalities can be employed, most local public health departments plan on using points of dispensing (PODs) as the primary means of MCM delivery [1]. There are two types of PODs: open and closed [2]. Open PODs are typically operated by local public health agencies and are available to the general public. In contrast, closed PODs are operated privately and are limited to smaller groups of people, such as government agencies, hospitals, long-term care facilities (LTCF), private businesses, faith-based organizations, and academic institutions [3].

In the U.S., state and local jurisdictions are responsible for rapid and comprehensive distribution of MCMs to the public [1, 4]. However, researchers indicate that many public health agencies lack the ability to conduct mass dispensing of MCMs through open PODs alone [5, 6]. Therefore, most public health agencies rely upon closed PODs to aid in MCM distribution, in order to alleviate burden on open PODs [6]. Furthermore, researchers have found that more extensive closed POD coverage through development of a closed POD network (i.e., a collection of closed POD sites representing businesses, agencies, and organizations from across the region), has been associated with increased perception of readiness to dispense MCMs to a community [5]. However, a closed POD network will only be effective if the participating closed POD sites are ready to deploy and dispense MCMs. After sites are recruited to be a closed POD, they need to be managed. Few studies have focused on how to assess closed POD preparedness and none have examined best practices for managing sites once they have been recruited. The purpose of this study was to identify U.S. disaster planners’ approaches, practices, and challenges to managing, sustaining, and assessing closed POD sites.

## Methods

Recruitment occurred from August—November, 2019, and involved research team members calling U.S. public health disaster planners designated as managing open and closed PODs at their agency. A jurisdiction must have had at least one closed POD site in order to be eligible to participate. Qualitative interviews occurred with willing participants; demographic data was also collected. The team aimed for 15 disaster planner participants; recruitment ended when sufficient interviews were scheduled. Participants were provided an electronic copy of the recruitment statement after they agreed to participate. Verbal consent was obtained prior to the interview being started by asking each participant if they had read the recruitment statement and agreed to participate. Interview questions consisted of eight items related to the frequency and types of communication between the health department and each site, training provided to each site, how and extent to which closed POD site readiness was assessed, management of non-progressing sites, and challenges faced in the closed POD management, evaluation, and sustainment processes. Participants were told that their data would be anonymous, unless they elected to publicly share documents. Interviews were audiotaped and then transcribed verbatim. The Saint Louis University Institutional Review Board approved this study with the methods described above (IRB protocol # 30417).

### Data analysis

Interview transcripts were coded using content-analysis to identify and categorize major themes. Participants’ quotes that exemplify the major themes are reported. Words or phrases in parentheses were added to clarify the quote and are not the participants’ actual words. Participants’ demographic data were analyzed using the Statistical Package for the Social Sciences (SPSS) 27.0.

## Results

In all, 19 disaster planners were asked to participate. One was ineligible due to not having any closed PODs; 16 public health disaster planners agreed and two elected not to participate. Participant and jurisdiction demographics are outlined in Table 1, as well as closed POD coverage per agency. Three (18.8%) were part of a tribal community; the remainder (81.3%, n = 13) were part of a Cities Readiness Initiative (CRI) jurisdiction (Table 1). About two thirds are in a jurisdiction with a population between 100,000–500,000 or 500,001–599,999 (31.3% and 37.5%, respectively); few (12.5%) were from the smallest or largest-sized jurisdictions (Table 1). Half (50.0%, n = 8) were male and aged 26–35 years; the remainder were 36–45 or 56 or older (each 25.0%; Table1). About a third had 2–5 years, 6–10 years, or 11 or more years of POD work experience (31.3%, 31.3%, and 37.5%, respectively; Table 1). Two thirds (62.6%) reported that their closed POD network covers 30% of less of their population (Table 1).

**Table 1:** Jurisdiction and participant demographics.

Jurisdiction Characteristic	All Respondents N = 16% (n)
**Part of a Cities Readiness Initiative (CRI) jurisdiction**	81.3 (13)
**Part of a Tribal Community**	18.8 (3)
**Population of Jurisdiction**	
≤ 100,000	12.5 (2)
100,001–500,000	31.3 (5)
500,001–999,999	37.5 (6)
1 million—2,499,999	18.8 (3)
**Geographical Region**	
West	37.5 (6)
Midwest	31.3 (5)
South	18.8 (3)
Northeast	12.5 (2)
**Percentage of Population Covered by a Closed POD**	
0–10%	31.3 (5)
11–30%	31.3 (5)
31–50%	25.0 (4)
≥ 51%	12.5 (2)
**Participant Characteristic**	**N = 16% (n)**
**Age**	
26–35 years	50.0 (8)
36–45 years	25.0 (4)
≥ 56 years	25.0 (4)
**Gender**	
Female	50.0 (8)
Male	50.0 (8)
**Years in Current Role**	
≤ 1 year	6.3 (1)
2–5 years	43.8 (7)
6–10 years	31.3 (5)
≥ 11 years	18.8 (3)
**Years of Work Experience in Field**	
2–5 years	31.3 (5)
6–10 years	31.3 (5)
≥ 11 years	37.5 (6)

A list of closed POD sites covered by the participating jurisdictions is outlined in Table 2. The number of closed POD sites at each jurisdiction ranged from 2–8, with an average of 6 sites per participating jurisdiction. Almost all (93.8%, n = 15) had a hospital(s) as a closed POD site (Table 2). Three quarters (n = 12) had at least one long-term care facility (LTCF) closed POD site (Table 2). Two-thirds had a closed POD site for first responders, private business(s), or non-healthcare business (Table 2).

**Table 2:** Current jurisdictional closed point of dispensing (POD) sites.

Organization or Group	Currently Covered by a Closed POD N = 16% (n)
**Hospital**	93.8 (15)
**Long-term care**	75.0 (12)
**First responders**	68.8 (11)
**Private business**	68.8 (11)
**Non-hospital healthcare agency**	68.8 (11)
**City/county, federal or state employees**	43.8 (7)
**Utilities or public transit**	25.0 (4)
**Faith-based or civic organizations**	25.0 (4)
**Academic institution (school or university)**	25.0 (4)
**U.S. Postal Service (USPS) personnel**	18.8 (3)

### Communication with closed POD sites

After a site has been established as a closed POD, health department officials maintain regular communications with their point(s)-of-contact at these sites. Though the frequency of communication varied, the disaster planners interviewed reported annual communication with their sites at a minimum, with many disaster planners communicating biannually, quarterly, and monthly. As one planner described, “once everybody’s on-board, then it’s just making sure that at least several times a year you’re contacting them, reaching out to them, making sure that there hasn’t been any changes, any staff turnover, changes to their point-of-contact who would actually activate their site.” A few disaster planners discussed the role of renewing MOUs as a way of communicating with sites and ensuring that they stay engaged and willing to participate. As one planner explained, “Most sites have a 3-year or 5-year MOU time limit. It kind of forces the county to go back and revisit the site and remind them that they’re a closed POD.”

For some, the amount of communication depended on the site’s enthusiasm or whether there is a POD exercise planned. As one disaster planner elaborated:

*[Frequency of communication] is a case- by-case basis. There’s some closed POD partners that just want that one-year bother to make sure the phone numbers are the same and then leave them alone unless people are dying of anthrax. And there’s other partners that are eager to stay connected. So, it varies on the number of interactions just based on a case-by-case basis.*

Modalities for communication between health departments and closed PODs included regularly scheduled emails or phone calls, newsletters, call-down drills, and face-to-face meetings. To keep communication with their sites, one disaster planner said, “If there is new information about closed PODs or PODs in general, I try to send updates and that can be…every few months or sometimes it ends up just being annually if things are really crazy hectic.” Other disaster planners use call-down drills to keep their sites engaged. “At a minimum, we have quarterly communications through our quarterly communication drills with all of our POD partners,” said a disaster planner.

### Training provided to new and established closed POD sites

Almost every participating public health official stated that their organization made the commitment to provide trainings and assist with exercises to ensure effective functioning of the closed PODs. A few public health officials required a commitment of attendance at either their annual or semi-annual training sessions and tabletop exercises in order for the closed POD to maintain active. However, the majority of disaster planners said that trainings and exercises were voluntary for the closed PODs. As one participant stated, “We encourage them to have an exercise, but it is not required.” Some exercises were even hosted by the closed POD site themselves, during which they would ask public health officials to come for support. Some exercises are used to help recruit new sites and illustrate the site’s responsibilities. As disaster planners explained:

*I recommend you have an exercise to include anybody who you think you would like to recruit. For example, when we did [an exercise with] our long term care centers, we invited the schools. The schools said, “This sounds pretty good. Maybe we should be a closed POD.” So I would just say, that if anybody wants to have somebody to be a closed POD, invite them to an exercise with ones that are already established and make sure you write them in [to the scenario]. For example, “this is what’s going to happen. This affects kids at a school, and on and on and on.” And suddenly whoever it is that you want to be a closed POD is drawn into it and they have to ask themselves just how would they do this. The schools who were involved, they said, “You know what, it sound like, like this would be something good for us to do.” And it is all because they were invited [to the exercise]*.

*We’ve actually used POD exercises to kind of help gateway some of those [LTCF] partners into the coalition and helped too to reach a few more people to recruit for closed PODs.*

The format and frequency of trainings varied based upon the coordinating public health official. Some described a standard list of training that they offer; others described a more informal process of training, depending on the site’s interests and needs. One participant stated, “We try to give them as much information and training as they are available to receive.” A list of POD trainings provided by participants is outlined in Table 3. In states that allow non-licensed/non-medical providers to hand out MCMs in PODs, dispensing training was frequently mentioned as essential to increase the number of staff able to assist in the POD. As one planner said, “We as certified trainers through the state can turn John Q public into a dispenser during an emergency, but it has to be a declared emergency. And then they can dispense pills.” In other states, this is not currently allowed, but is under consideration. As one planner explained, “Right now, the state and the board of pharmacy are trying to see what they can do so that we could have more of a workforce [dispense MCMs], because it’s a completely medical model right now. It would have to be a licensed professional as it is now, but the state and the board of pharmacy are looking into changing that.”

**Table 3:** Examples of content or types of trainings provided by disaster planners to closed pod site staff.

Content or Type of Training
How to develop a closed POD plan
POD Essentials [formal training that covers greeting, screening, triage, dispensing; responder wellness]
MCM dispensing training for non-licensed/non-medical individuals
POD operations; possible POD layouts; how many staff are needed; how they receive medications; how to manage disruption/maintain security
POD roles and responsibilities; when and why it would deploy
Legal issues related to PODs

MCM = medical countermeasure; POD = point of dispensing

Training topics were reported to vary, depending upon the interest or need among the closed POD site staff, such as whether it was a new or existing site. Early training for new sites often included education about how to develop a closed POD plan. One disaster planner mentioned that their health department only offers written training materials, but the rest all offered virtual or on-site training on a once-on-one or group basis. Many offered train-the-trainer educational options. On-site training was often prioritized as a way of building partnership and collaboration with closed POD sites or when a closed POD administrator or other staff position would turnover. As disaster planners explained:

*I can’t compliment [my sites] enough on their willingness to cooperate with us, their willingness to be closed PODs. And I think that a big part of that is because I take the time to talk to them face-to-face and the more often you could do that, the more they feel like they are part of us, part of a team. And when it comes down to another event that hits or something that would require the SNS [Strategic National Stockpile], we’ve got to work together or we can’t get accomplished what we need to accomplish*.

*I have PODs that want [onsite training] done, not necessarily annually, but anytime that they have like kind of some significant staff changes where they need to be kind of retrained for those individuals. Some actually prefer onsite training, because maybe the main admin person has been the same, but they have higher turnover with the other staff in the structure for the closed POD*.

*I’ve had a facility request for me to go to their facility, just so they can show me and ask me questions about where they are thinking about setting up the POD and how the flow would go.*

Insights and comments made by disaster planners about their approach to closed POD training and exercises:

*We usually only do [the training] when they enroll, and then we contact them again, like every year. We always say like, “if you need this training again, reach out to us.” And I have done re-training to our closed PODs that have requested it*.

*[The closed POD site] can invite whomever [to the training]. It could be just me and that administrator, if they want me to talk through the whole process that they have in place already, or sometimes they invite me at a time when they have an all staff meeting so then I can just do the training right then and there with them*.

*I work with [the site] to plan a tabletop exercise where we bring in the leadership and talk through some of the scenarios. And I think that helps people get a better understanding of the why’s and how’s of a closed POD*.

*We usually try to do the first exercise quickly after they join, within a quarter, within three months. And I like to make it like a training-exercise thing, so we are talking through and coaching as we are doing this table discussion. And then we go from that within, hopefully, the next month after that, to testing it, and then after that it will become an annual thing*.

*Our drills are done quarterly, and they typically are done between Monday and Thursday evening around 6:45 in the evening. That’s the sweet spot where it’s after duty hours, but you’re not really inconveniencing too many people. Most people have already eaten dinner, but they haven’t sat down to watch their favorite TV shows.*

Participants indicated that most POD exercises are held with the same goal: to prepare the closed POD for deployment. These trainings are meant to demonstrate that the closed POD site can deploy successfully, independent of local public health. Public health disaster planners attend every training and exercise possible to help guide and prepare the site for deployment, but they make it clear to the site that they will not be available during an actual event. Ultimately, the closed PODs are recruited to “reduce the burden on the health department at [open POD] operations.” Disaster planners in rural areas emphasized the need for regional POD exercises to aid in deployment readiness, but also to collaborate with other sites on best practices. As rural disaster planners explained:

*Especially because we are rural, we work with our other neighboring health departments and other emergency response coordinators in our region and across the state. We share ideas and exercises and evaluation roles and things across our jurisdictions. And so, I think it helps you to work with your neighbors, for things that we might do differently or ways to approach a problem or a barrier*.

*We have only a couple regional EOC meetings; there’s 20 districts throughout the state. But there are four of us who worked together a lot and we are evaluators at each other’s place. So what happens is, as an evaluator I might see something that I can recommend, because their place is different. They have different long term care centers, different hospitals, schools, and how they approach things, and so by working together we’re able to get information from each other, get suggestions from each other.*

### Assessing closed POD site readiness to deploy

Most disaster planners described their POD readiness assessment process as being informal or non-existent. As one disaster planner explained, “I don’t have a regular formal process of going to the site and assessing them for whether or not they could deploy, but I do assess their plan early on. It just involves looking at their written plan.” As another explained, “We don’t do a site assessment, because they agree to the site responsibilities when they sign off on the MOU that they have a space and they agree to the staffing.” Some public health officials do not even require sites to have a written plan. As one disaster planner explained, “We highly recommend that they do a training and that they have a specific site plan based on the layout of the room or wherever they are going to do it, but we don’t require them to come up with a plan.”

Many disaster planners described using exercises to assess site readiness to deploy in addition to being a form of training. However, though disaster planners use exercises to assess deployment readiness, none described a formal process for evaluating site performance during exercises. It appears a qualitative assessment process is being used. The following are quotes from disaster planners regarding their use of exercises to assess site readiness:

*We set [the exercise] up like they were actually going to [deploy] and see if there’s anything in that process that they need to change. Sometimes visual is much easier to think through all the what-ifs and the possibilities that might happen, more than just words on paper. So, I would definitely advocate for [exercises]. I think that is an important element to determine whether or not they’re truly ready to activate*.

*Once they have a plan and they’ve received some training, we use the drill or the POD exercise to assess their ability to [deploy]*.

*On an annual basis, we have certain exercises that need to get done, so like a facilities set up and site activation [drill]…and we’ll sort of rotate through our different sites so that we’re not exercising the same procedures every year with the same site.*

Some disaster planners had a formal process to evaluate site readiness to deploy. For example, one disaster planned said, “It comes down to what they have documented in their plan. I review all of their plans.” Another disaster planner said they select a sample of their closed POD sites each year and conduct an on-site evaluation using a checklist of preparedness items, such as whether they have a written plan, POD roles assigned, job action sheets for each position, and whether they have conducted an exercise in the past year. As the planner explained, “It’s like an annual assessment where we’ll call them up, make an appointment, send members of our office out to conduct the assessment. Sometimes we track the results on little charts and things, but usually it’s just kind of a way of keeping our fingers on the pulse and seeing who needs help out there.” According to the participating disaster planner, this assessment can be completed in about 20 minutes.

### Managing closed POD sites that are not participating or making progress

Disaster planners took differing stances on how to manage sites that are not actively participating in POD planning and/or not making progress towards improving their readiness to deploy. A few planners had not experienced this at all. They indicated that all of their sites were actively engaged and appeared to be making progress, but this was the exception. Many disaster planners described the challenges in managing sites that are not making progress, but they hesitate to remove a site from their closed POD network or regional collection of closed POD sites. Some planners indicated that they would only terminate a closed POD site if the site requested it or if they lost the ability to dispense medications. As one disaster planner explained, “We normally just remove them if they’ve requested it or if we just don’t hear back from them during our yearly check-ins, then we’ll remove them.” As another explained, “We typically won’t remove [a site] just because we don’t think that they’re deployable, because it’s their determination [if they can be successful]. They’re not required to deploy.” Some planners pointed out that staff turnover could necessitate removing a site from being a POD: “My state requires MDs [physicians], PAs [physician assistants], or APRNs [advanced practice nurses] to actually dispense [MCMs]. If something changes where [the site] either loses that role or that department is now gone and has not been refilled, that would make us terminate a closed POD agreement because it would be going against state statute.”

Rather than removing a site as a closed POD partner, most disaster planners discussed approaches to re-engaging sites in the closed POD planning process. This consisted primarily of contacting the site’s point-of-contact and inquiring as to why progress is not being made. As disaster planners explained:

*I would go in there and see like why [they aren’t making progress]. What is the hold up? I don’t want to do everything for them, you know, but when it comes down to get the ball rolling, it would be what is the reason why. Is it just that everyone is overworked right now, and you just don’t have the time to do it or do you not want to do it?*

*Yeah so, I would intervene [if they weren’t making progress], because it’s a small enough community. We talk to them, we call them. If they’re not making any plans, see if they are still willing or into it. And I’ve helped them, you know. Gone back and done another training just informally with one site and helped them write the plan as we go*.

*We can sense whether the enthusiasm is waning or maybe there is pressure being applied to not be a part of the program anymore. Or maybe the person who is the delegated POD guru just doesn’t have the time. And so then I usually say, “You know, this program is completely voluntary, and you can withdraw at any time. But if there is any help that you need, let me know” and I will usually give them my card again and they have my contact information. I say, “Don’t hesitate to call. We can come out here. We can help you. We can look at your plan. We can figure out the way that you can get other people involved in this.”*

A few disaster planners took a firmer stance and stated that they would remove a site if the site was not making progress and/or not participating in the planning process. The primary indicator of a site not making progress was a lack of engagement/participation. As one disaster planning explained, “We hate to remove [a site], but if you are not showing your face year after year, and not coming to trainings, not coming to actual deployments, then what are you doing [in our closed POD network]?” As another explained, “If they have a plan, but they are not showing up to coalition meetings, they are not doing exercises on it, they are not communicating back and forth, then I would have no choice but to say ok this facility clearly does not want to be a closed POD anymore.” A third explained, “For sure we drop them if they are not keeping up communication, because I have had some that have had to drop off because they are not returning the updated annual form like we want.” One said that they remove non-participating sites in order to save their limited work time focusing on sites that are engaged. As they explained, “I tell them, ‘You can always come back, but without any progress, we don’t really have the bandwidth to keep you on without willingness.’ I’m not a big fan of having numbers on the books, just for having numbers on the books.” Another indicated that they remove non-participating sites, so that they are not wasting MCMs on a site that is unable to deploy. As they explained, “I don’t want to give them the MCMs, because I don’t think they could actually deploy and distribute those. I would rather have those individuals go to an open POD.” One disaster planner mentioned that they remove sites from their POD list if the site’s MOU expired 6 months or more ago.

Although some disaster planners discussed how and why they would remove a site from their closed POD network, all of them emphasized that this would only be done as a last resort. They described the numerous attempts they would make to keep all sites active, including calling, emailing, and/or visiting the site multiple times. When disaster planners had been able to reach someone at the site to inquire about why they are no longer engaged, the most common reason provided by sites was a change in staff. As one planner explained, “A lot of it is just due to turnover in the facility and the new person that has come in is just not interested in continuing as a closed POD.” Although some sites admitted to not wanting to stay involved, it was more common for a site to simply stop communicating with the health department, and the disaster planner eventually stopped trying to reach out, assumed they no longer want to be involved, and removed them from the closed POD site list.

### Managing healthcare versus non-healthcare-based closed POD sites

Most disaster planners indicated that they manage hospitals and healthcare closed POD sites differently than they manage non-healthcare sites. These differences ranged from the frequency of communication to the type of training provided to the sites. For example, a few disaster planners noted that they communicate more often with healthcare sites compared to non-healthcare sites due to organic meetings related to non-POD-related issues, such as healthcare coalition meetings. As one planner noted, “With our healthcare coalition, we communicate with those partners very regularly. At least once a month if not more than that. With the [non-healthcare] businesses, I would say communication is more limited with them.” One disaster planner said that they do not require an MOU from their hospital partners, but do with all of their non-healthcare closed POD sites. As the planner explained, “All hospitals in [their jurisdiction] are considered closed PODs, but we don’t require an agreement although we do require them to have an approved plan.”

Disaster planners discussed their different approaches to training at a healthcare agency versus a non-healthcare business. They recommended providing traditional training to non-healthcare businesses, but consider using a combination of train-the-trainer and just-in-time training approach for healthcare agencies, especially LTCFs. This is because most healthcare facilities are unable to have a large number of staff available for training at the same time unless the training is provided before or after a shift starts. As disaster planners explained,

*It’s tough to get all staff to a training, especially at long term care facilities. Businesses will definitely be easier because they can just say, ‘hey stop what you are doing for today and come to this training’. But long-term care facilities have to run as usual, so pulling out all staff who can act as vaccinators is tough. We have to rely on going to their lead, the floor managers, or the clinical services managers and have them relay that information to their staff*.

*A lot of healthcare and LTCF staff are not going to have much training prior to [POD deployment]. It’s really going to be the just-in-time training that’s provided as well as when we do exercises. We will be doing tabletop exercises as well as the full-scale drill, which will kind of act as their training*.

*Businesses need more training, more set up than a long-term care facility, because LTCFs are used to giving medication, used to giving vaccines to residents and family members. A business is kind of going to need a lot more like coaching along through the process, you know help with the set-up of the plan and especially with exercises. Because businesses, aside from their fire alarms that are required like from OSHA and things of that nature, this will be all new to them completely.*

Many disaster planners described healthcare sites as being easier to manage than non-healthcare POD sites. As disaster planners explained:

*I can trust a healthcare site more than I can trust a non-medical or non-healthcare site to take care of, you know, dispensing type of operations. From a healthcare standpoint, the staff are highly qualified, they’re already trained and they actually know how to dispense and/or vaccinate, so there’s less for me as a planner to have to worry about providing detailed training relative to those tasks. They are also a little more eager to help us out and get through this process. They understand the mission, they understand wanting to help people, and wanting to prevent people from becoming ill, so I think that lends them to be great closed POD entities*.

*I would say that [healthcare agencies] are better prepared because they’re required to do the flu shot every year, so they are able to exercise their plans. And they work more closely with the state department of health on receiving treatment supplies from the SNS, so they also have that knowledge of like, if this were a declared emergency, this is why this is happening. I feel like some of our other private organizations, even though they understand that it’s a benefit for their productivity and continuity of operations, they still don’t get the big picture of why this is important. But the hospitals get it*.

*Healthcare facilities are easier because I see them more regularly and they had the requirement from the Centers for Medicare & Medicaid Services (CMS) as well as licensing and certification from our state. So, they have requirements for emergency preparedness and they see the need and they see the requirements. On the business side, there is no regulation for that, so it takes a lot for us to convince them why they should be a closed POD partner, why they should communicate with us. Like why it is important for them to have emergency plans and procedures in place*.

*Hospitals and clinics, they know what they’re doing. They exercise and they have practices in place. So we’re more hands off on with them and provide them the vaccine or the MCMs, but they really run their own thing. We’re really more hands on and give a little bit more guidance with those sites that are non-medical.*

One disaster planner noted that hospitals are better engaged than LTCFs. As this planner explained, “I’d say our home health agencies are our hardest ones to keep engaged. Usually during call downs, they are the hardest ones to get ahold of. When we are having the meetings, they are the last that we hear from. We call them numerous times to try to get ahold of somebody. I think it could be they don’t have a lot of staff and so they are out running around.”

### Challenges to managing closed POD sites

Many disaster planners reported that their largest challenge in managing closed POD sites relates to frequent staff turnover, both within public health and at the closed POD sites. Many disaster planners discussed high turnover of public health staff involved in POD preparedness that has led to difficulty managing existing closed PODs. As one disaster planner said:

*If you want to count the last 5 years, every year we’ve had a different MCM coordinator. So um, with every new MCM coordinator that comes on in our county, they try to start up the partnership agreements again. It probably contributes to the fact that we are having a hard time with the businesses, because even on our end, we’ve had a lot of turnover.*

Staff turnover at the closed POD site had also been a major challenge. Points of contact often changed due to individuals shifting positions or leaving entirely, which makes communication difficult and can stall preparedness efforts. Often, staff left without anyone notifying the disaster planner, resulting in inaccurate information for a point of contact at a site. To remedy this, many disaster planners conducted frequent check-ins to ensure the contact person/people at the closed POD was correct. One planner explained, “We update the point of contact every 6 months, because we do a [rapid electronic notification system] drill every 6 months and so…we reach out to each agency, and say, ‘Is this still the correct contact information for your facility?’”

Staff turnover even resulted in a site voluntarily withdrawing from the closed POD program. Planners described scenarios in which a change in personnel led to a site leaving or almost leaving the closed POD network:

*If they don’t respond back, usually within a week with their annual update, I’ll try to give them a call, send the email again, things like that. A lot of times it comes back returned, undeliverable, because it’s maybe a new person. If it does come back undeliverable, I do try to contact the facility or stop by and visit to see if there is a new person, and then go from there…and then there have been new people and they have said, “No, I’m not interested in continuing [to be a closed POD].”*

*Our MOUs state that they will automatically renew unless we receive written documentation that they don’t want it to. So, one organization in particular had an MOU signed back in 2009. They had lots of turnover since then. The new COO [Chief Operating Officer] had no idea this MOU was signed for this organization, and had no idea that they were a closed POD. So, it kinda started the whole re-recruitment cycle over again.*

Disaster planners frequently cited a drop-off in communication as a challenge for maintaining a closed POD network. As one planner explained, “Our biggest concern with these sites is communication. We really don’t want any sites to kind of go rogue, so to speak”. Another noted that the sites often did not tell public health about their staff turnovers; it needed to be discovered by the disaster planner. As the planner explained, “I’d say probably a good 75–80% of the time we reach out to them because we’ve heard of [the point-of-contact leaving]. Sometimes, the good ones reach out to us…those that are a little bit more active. But most of the time, it’s us reaching out to them because we heard or because we did a call down drill and we realized that someone wasn’t there and the contact information needed changed.” The lack of communication and participation has led some disaster planners to question whether a site will be successful if it needed to deploy. As one disaster planner explained, “We do quarterly recall drills, and typically the response rate hovers around 65% so it’s not great. And that leads me to believe that in a real event, we are not going to be able to rely on a lot of these organizations that we’ve put some time and effort in to recruit and give information.”

## Discussion

Closed POD management is vital to maintaining a POD network capable of deployment. Findings from this study indicate that closed POD management is a time-consuming and challenging endeavor. Management of a closed POD network begins with site recruitment, but requires significant ongoing investment of human capital in terms of public health for maintenance. Components of a closed POD management program include regular communication with sites, initial and ongoing training, and assessment of deployment readiness through plan review, exercises, or other formal processes. A comprehensive maintenance program maximizes the chances for successful rapid POD mobilization [6].

Some of the biggest challenges in POD management identified in this study included closed POD site staff turnover and lack of site engagement. Participating disaster planners noted that frequent communication and being proactive are essential. Two best practices identified in this study were to conduct frequent check-ins with sites to ensure that the contact list is up-to-date and building partnerships with closed POD site personnel. Doing so keeps the site engaged and allows the disaster planner to be informed about staff turnover changes that could affect the site’s participation in the closed POD program. Staff turnover has been reported as being an obstacle to open POD preparedness [5]; therefore, it is not surprising that it would negatively affect closed POD preparedness as well. In addition, staff turnover is more common in LTCFs compared to other types of healthcare agencies [7, 8], which may make maintaining LTCF closed POD sites extremely challenging. Building partnerships between public health disaster planners and closed POD site points-of-contact has been identified as a best practice for keeping sites engaged.

Disaster planners in this study noted that training was necessary both for establishing a closed POD site as well maintaining and improving preparedness over time. Though training requires an ongoing time investment from disaster planners, these educational programs ultimately serve to reduce the burden on the health department by maximizing the ability of closed PODs to function independently [4]. Though participating disaster planners in this study all described varying training programs they offer, prior studies have found that little to no pre-event training is being provided to closed POD staff [4, 9]. This could reflect improvements in closed POD management practices over time or the planners in this study may be providing a more robust preparedness program compared to other jurisdictions. Further improvements in training are recommended, including the need to utilize a more standardized regional approach, allowing closed PODs to work together and establish best practices [10]. The need for standardized training is proving necessary with the current wide-scale deployment of COVID-19 vaccines [11]. Beyond COVID-19, however, there are threats that could result in POD deployment, necessitating a trained workforce ready to dispense oral or injectable MCMs within a brief time period [10]. Training for this process is essential [2, 12].

An essential and challenging component of closed POD management identified in this study was the use of drills and exercises. Drills have been previously noted as an important method of ensuring preparedness, specifically to aid in improvement planning [3, 13]. In previous studies, both open and closed PODs have been found to only engage in exercises infrequently [3, 5, 13, 14]. For example, a 2017 study [3] found that fewer than a third of all closed POD sites in one jurisdiction had conducted a drill in the past two years. In this study, though disaster planners frequently discussed implementing drills with closed POD sites, they did so in relation to providing training rather than using exercises to assess deployment readiness. In addition, none of the participating planners in this study nor any previous research have described conducting closed POD exercises aimed at assessing the ability to perform repeat visits. Repeat visits will be required for multi-dose vaccines, such as anthrax and the current COVID-19 vaccines, or regimens of oral antimicrobials if it is not distributed during a single visit. Preparation for repeat visits should be incorporated into closed POD exercises because they pose unique challenges. Puerini et al. [15] assessed POD throughput for scenarios involving a second visit and enhanced screening, and provided estimates in terms of staff and resources required. Most notably, they identified the need to balance the time required for enhanced screening to differentiate MCM side effects from possible infection with the need to have high throughput [15]. This will be a challenge during administration of the first COVID-19 vaccines, because they are known to have side effects that may create a more prolonged discussion and screening for those coming in for their second dose [16]. POD preparedness will also include the need to have a communication system in place to notify and/or remind individuals when it is time for their second or subsequent doses [17]. Text reminder systems have been found to be an effective method of increasing compliance with second doses of vaccine [17, 18]. Additionally a unique challenge identified in COVID-19 vaccine rollout has been the difficulty of reaching the high-risk elderly population who may not be engaged in technology-based signup and reminder systems, such as websites and text messaging. This difficulty is compounded by the ongoing delays in postal service delivery, limiting some alternative options to reach the community [19].

One area for improvement identified through this study related to site assessment. Most disaster planners in this study described using an informal process for site assessment, which could result in sites being unable to deploy successfully. The CDC [1], Federal Emergency Management Agency (FEMA) [20], and U.S. Homeland Security Exercise and Evaluation Program (HSEEP) [21] all indicate that formal assessments are necessary to ensure preparedness. At the federal level, the CDC requires periodic formal site assessments using the Operational Readiness Review (ORR) program [22]. The ORR includes a section about closed PODs, but it is a broad assessment tool that consists primarily of an accounting of how many closed POD sites exist and are assessed each year by the jurisdiction versus outlining clear preparedness indicators that could be used as part of an assessment. There is currently no standardized tool for assessing closed PODs. Participating disaster planners who reported conducting formal assessments primarily used exercises as the assessment method, although a few discussed reviewing POD plans. In addition, one identified a formal process using an on-site or phone-based annual review. This planner noted that the process is not resource consuming, as it can be done in less than 30 minutes. Formal assessments are necessary to validate plans, protocols, and throughput capabilities; they also help identify areas for improvement. In addition, a 2017 study found that closed POD sites that had been formally assessed more frequently performed better in the objective preparedness assessment [3]. The lack of a clear assessment process expressed by most participating disaster planners could result in failure of the closed POD when time to deploy. Disaster planners should aim to incorporate formal assessments into their closed POD programs to determine site deployment readiness and increase communication between the site and public health officials. The disaster planner who described having a formal assessment tool has graciously agreed to share their instrument. Readers interested in obtaining a copy of the assessment tool may email the primary author.

An ongoing challenge identified through this study relates to managing sites that do not appear to be engaged in the planning process. It is unknown whether these sites would actually be able to deploy successfully when needed. Despite this, participating disaster planners often expressed hesitancy to remove a site, even after repeated attempts at reengagement. This may be partially due to the amount of effort needed to recruit new sites. A formal assessment tool could be useful in initiating discussions with struggling POD sites in an attempt to improve their preparedness and increase engagement. In this study, a complete lack of engagement or communication was identified as the trigger for elimination as a POD site, and that removal was commonly done passively without a formal removal communication. Engaging in more frequent and standardized communication methods may improve retention of closed POD sites. In addition, frequent check-ins with the site point-of-contact may better ensure that the health department is aware of updated staffing changes so that communications do not go unanswered. An upfront investment in time communicating with existing closed POD sites may save the disaster planner the longer term need to recruit additional or new sites.

An interesting finding from this study was that many disaster planners manage healthcare-based closed PODs differently than non-healthcare sites, including having more frequent communication with them. In many cases, this was due to the established relationships between the healthcare site and the health department through healthcare coalitions. However, although disaster planners reported increased communication with healthcare sites, many noted that they offer less training to these sites. This was due to staffing challenges in LTCFs and a perception of MCM distribution being more similar to the regular activity of a healthcare site compared to a non-healthcare business or organization. Despite this, a previous national study found that LTCFs were significantly less prepared than other types of closed POD sites, including hospitals and other healthcare agencies [3]. In addition, LTCFs were found to have less access to personal protective equipment (PPE) and lacked infection prevention guidance during the COVID-19 pandemic [23]. These gaps were due to prioritizing PPE for acute care settings versus LTCFs and a general lack of trained infection preventionists working in LTCFs that have existed across healthcare settings even prior to the COVID-19 pandemic [23, 24]. Therefore, it may not be prudent for disaster planners to leave healthcare sites out of closed POD training. Closed POD preparedness is essential across all healthcare POD sites, especially LTCFs that have a vulnerable population who would have difficulty accessing an open POD site. Evidence of LTCF vulnerability during events requiring mass MCM distribution is currently illustrated through the initial roll out of COVID-19 vaccine and the need to prioritize vaccination among LTCF residents and staff [25, 26]. Public health disaster planners might consider investing more time and effort in non-healthcare sites to get them ready for deployment, but should not neglect healthcare sites, especially LTCFs that will be crucial to successful MCM delivery during a future event. One critical component of LTCF staff training that has been identified is the need for ongoing infection prevention and control education, which has historically only been provided to staff when hired [27]. This would be essential as part of LTCFs’ closed POD training because the site would need to limit disease transmission as they were dispensing MCMs to their patients.

This is the first study to examine extensively current and best practices related to closed POD site management. Participating disaster planners provided robust and detailed information about their closed POD management practices and challenges. However, limitations should also be noted. Although this was a national study, the sample size was small because it was a qualitative study. A national quantitative survey may have elicited different findings and would likely be more generalizable. Self-selection bias may have affected the findings; participants’ experiences may have differed significantly from non-participating jurisdictions. For example, it is likely that participating public health professionals have more experience with closed PODs compared to those who elected not to participate. Newer or less experienced public health professionals would likely have expressed different challenges. Lastly, data collection occurred in fall, 2019, immediately prior to the start of the COVID-19 pandemic. It is highly likely that participants’ closed POD management practices had to be decreased or even eliminated during some or all of the pandemic, though this is not known.

## Conclusion

The work of closed POD management does not end with recruitment and requires robust communication, standardized training, and formal assessment, all of which are frequently lacking in current closed POD programs. The primary challenges planners identified in this study included staff turnover and engagement, which require proactive and ongoing communication after recruitment. In addition, formalized assessment processes and protocols need to be implemented to ensure sites are ready to deploy rapidly and robustly when needed.
